# Telerobotic-assisted bone-drilling system using bilateral control with feed operation scaling and cutting force scaling

**DOI:** 10.1002/rcs.457

**Published:** 2012-01-24

**Authors:** Yusuke Kasahara, Hiromasa Kawana, Shin Usuda, Kouhei Ohnishi

**Affiliations:** 1 Department of System Design Engineering, Keio UniversityYokohama, Japan; 2 Department of Dentistry and Oral Surgery, Keio UniversityTokyo, Japan

**Keywords:** haptics, teleoperation, drilling system, bilateral control

## Abstract

**Background:**

Drilling is used in the medical field, especially in oral surgery and orthopaedics. In recent years, oral surgery involving dental implants has become more common. However, the risky drilling process causes serious accidents. To prevent these accidents, supporting systems such as robotic drilling systems are required.

**Methods:**

A telerobotic-assisted drilling system is proposed. An acceleration-based four-channel bilateral control system is implemented in linear actuators in a master–slave system for drill feeding. A reaction force observer is used instead of a force sensor for measuring cutting force. Cutting force transmits from a cutting material to a surgeon, who may feel a static cutting resistance force and vigorous cutting vibrations, via the master–slave system. Moreover, position scaling and force scaling are achieved. Scaling functions are used to achieve precise drilling and hazard detection via force sensation.

**Results:**

Cutting accuracy and reproducibility of the cutting force were evaluated by angular velocity/position error and frequency analysis of the cutting force, respectively, and errors were > 2.0 rpm and > 0.2 mm, respectively. Spectrum peaks of the cutting vibration were at the theoretical vibration frequencies of 30, 60 and 90 Hz.

**Conclusion**s**:**

The proposed telerobotic-assisted drilling system achieved precise manipulation of the drill feed and vivid feedback from the cutting force. Copyright © 2012 John Wiley & Sons, Ltd.

## Introduction

Drilling has a variety of applications. It is commonly used in the medical field, especially in oral surgery and orthopaedics. In recent years, dental implant surgeries have been focused upon and have been widely carried out 1. Drilling, which is the most important process in implant surgery, has been widely researched. Drilling shapes the holes for the insertion of dental implants. Precise manipulation of the drilling handpiece is required by the surgeon to avoid accidents. The most serious accident caused by imprecise manipulation of the handpiece is damage to the nerves, which leads to paralysis of the face. Thus, the quality of an operation mostly depends on the skill of the surgeon. Therefore, it is necessary to compensate for any shortcomings in the skill of medical care among surgeons to protect patients from medical accidents, and research and development of technology that will aid surgeons is required to prevent medical accidents.

In previous research, an image-guided supporting system 2 and a computer-aided training system 3–4 were proposed. In the case of the image-guided supporting system, radiography, computer tomography (CT) and digital volume tomography are often used for the planning and confirmation of the surgery plan. An advanced image-guided supporting system uses three-dimensional (3D) images of a skeleton; these images are constructed from a series of radiographs obtained using computer graphics in virtual space 5–6. In addition, in a current clinical test, telenavigation using a video-conferencing system and real-time sharing of 3D images over the internet was reported 7. However, the image-guided supporting system cannot yet visualize the internal structure of a bone. Hence, it is difficult for a surgeon to use drilling when performing surgery. In the case of computer-aided training systems, a surgery simulator incorporating haptic devices was proposed 8–9. This training system enables a trainee to virtually perform dental procedures by providing realistic tactile sensations in the same manner as they would do in the real world. However, these devices are not able to provide any assistance to real surgery.

One of the advantages of the teleoperated surgery system is the ability to perform tasks that exceed human dexterity. For instance, various teleoperated surgery systems were developed because scaling of motion was effective in the case of a minimally invasive surgery 10–11. Khan *et al*. proposed a position/force-scaled bilateral system that achieves micro-/nanomanipulation 16. An algorithm to detect surgical accidents by enhancing force feedback using a bilateral forceps system has been proposed 17. The algorithm denotes that the miniaturization of motion and the enhancement of the tactile sensation contribute to achieving difficult surgery with a minimum number of accidents. These facts indicate that the teleoperated surgery system is a useful technique for achieving a subtle motion of dental implant surgery. In this paper, a novel robotic drilling system using acceleration-based bilateral control is proposed for assisting real surgery. The purpose of this study was to examine whether the proposed teleoperated surgery system is useful.

The da Vinci, the most famous teleoperated minimal invasive surgery system, has been developed. However, several clinical reports indicate that it is necessary for a surgeon to feel the force of the drill in the teleoperated surgery system 12–13. Tactile sensations are important for a surgeon to check the condition of a patient. The importance of tactile sensations is common to minimally invasive surgery and implant surgery. Some teleoperated minimally invasive surgery systems with haptics have been developed in subsequent studies 14–15. Lawrence defined transparency in various bilateral control-type systems 18. Transparency is the criterion for evaluating the reproducibility of the contact environment and the operationality of the system. Yelmaz *et al*. proposed an optimized passive haptic robotic arm in computer-generated virtual implant surgery 19. The link length was designed to achieve the optimal robotic arm, based on transparency. Iida *et al*. proposed an acceleration-based bilateral control designed to achieve ideal reproducibility 20. In order to achieve teleoperation control with haptics, it is necessary to implement control of the position and force on the same axis. However, position control and force control must be implemented orthogonally on an axis in conventional hybrid control systems 21. The acceleration-based bilateral control system achieved position control and force control on the same axis without any interference during bilateral control. An acceleration-based bilateral control system is implemented in the proposed system to transmit a vivid cutting force.

A previous study reported the development of a robotic drilling system for dental implant surgery 22. This conventional system was a unilateral system, which was assumed to be applied to image-guided system. In these conventional systems, the contact force was not transmitted to the surgeon. In this study, the conventional system is enhanced to a master–slave system by transmitting the cutting force to the surgeon, using acceleration-based bilateral control. The scaling of the position force is implemented on the proposed system. The surgeon is able to cut the bone tissue precisely by feeling the cutting force through the proposed system.

This paper describes the structure of the proposed system, the acceleration-based bilateral control system and the experimental results, using a pig's lower jaw bone to evaluate the accuracy of position tracking and reproducibility of the cutting force.

## Materials and Methods

In this section, the proposed drilling system is introduced. First, the concept of the proposed drilling system is described. Then, angular velocity control and bilateral control are introduced.

### Telerobotic-assisted drilling system

**Figure 1:**
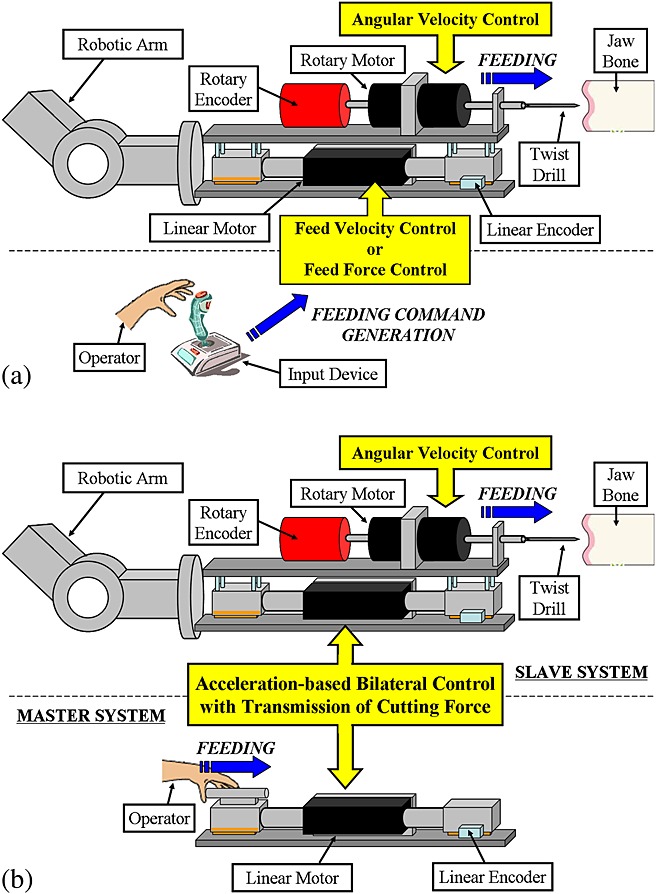
Structure of teleoperated drilling system: (a) conventional system; (b) proposed system

Figure 1 shows the difference in the structure of the conventional and proposed teleoperated drilling systems. The robotic drilling system typically consists of a rotary system and a feed system. The rotary system is composed of an electronic rotary motor and a rotary encoder. The twist drill is attached to the tip of the rotary motor. The drill rotation is kept constant by the rotary motor with the angular velocity control. The angular velocity response is calculated by pseudo-derivation of the angular response, which is measured by the rotary encoder. Pseudo-derivation is a numerical derivation combined with a low-pass filter (LPF) to eliminate the amplified high-frequency noise. The rotary system is connected mechanically to the feed system. In addition, the feed system is connected mechanically to a robotic arm, which is used for the positioning and fixing of the robotic drilling system.

The feed system is composed of a linear motor and a linear encoder. The conventional feed system selects the feed velocity control and the feed force control according to the intended use (Figure 1a). The feed system receives the feed velocity or force command from the operator using a joystick.

As seen in Figure 1b, the drill feed operation is manipulated by the operator through the master–slave system with the acceleration-based four-channel bilateral control, which achieves position tracking and the artificial law of action and reaction between the master system and the slave system. Therefore, the operator manipulates the drill feed operation by manipulating the master system. When the drill tip makes contact with the bone tissue, the cutting force is experienced by the slave system and is transmitted to the operator through the master system.

In the case of a general force control system, it is necessary to attach a force sensor to an end effector. However, this is not always possible because of the limitation in size of the end effector. In order to measure the force without a force sensor, the force responses need to be estimated using a reaction force observer (RFOB) 23. As a result, real-time monitoring of the cutting force can be achieved without any force sensors.

### Angular velocity control

**Figure 2:**
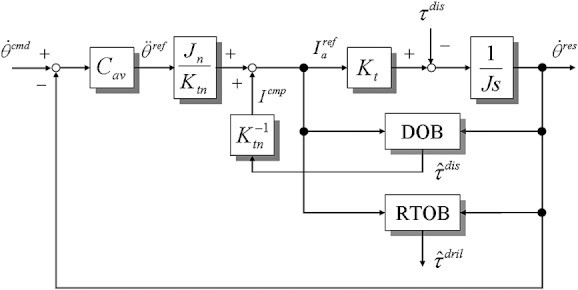
Angular velocity control

Figure 2 shows a block diagram of an angular velocity control system. The dynamics of the rotary system is obtained as:


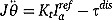


where *J* is the inertia, 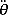 is the angular acceleration, *K*_*t*_ is the current-torque constant, 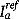 is the current reference, and *τ*^*dis*^ is the disturbance torque. The angular acceleration reference 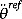 is obtained as:


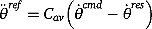


where *C*_*av*_ is the angular velocity controller, 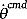 is the angular velocity command, and 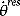 is the angular velocity response. The angular velocity controller *C*_*av*_ was a PI controller and is obtained by:


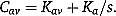


**Figure 3:**
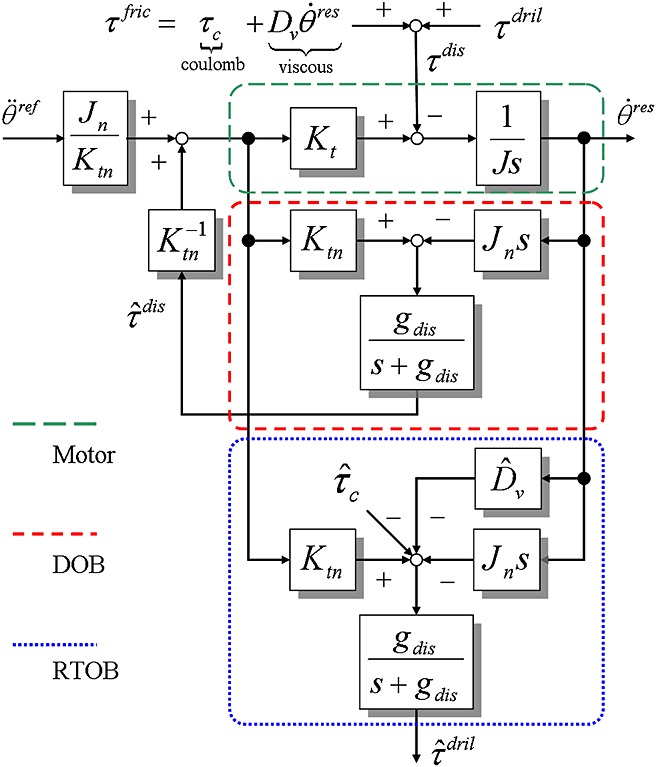
Acceleration control system

The nominal inertia *J*_*n*_ is obtained from the following equation:


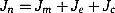


where *J*_*m*_ is the inertia of the rotary motor, *J*_*e*_ is the inertia of the rotary encoder and *J*_*c*_ is the inertia of the connecting parts between the encoder, motor and drill. Figure 3 shows a detailed diagram of a disturbance observer (DOB) and reaction torque observer (RTOB). The DOB-based system is a two-degree-of-freedom (DoF) control system known as an input-to-output and disturbance-to-output independent controller system. Robust motion control is achieved by using the DOB-based system 24. In the acceleration control system, the disturbance torque *τ*^*dis*^ is estimated by DOB as:


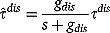


where 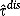 is the estimated disturbance torque, *g*_*dis*_ is the cut-off frequency of the DOB, and *s* is the Laplace operator. Acceleration control is necessary for achieving force control with a large bandwidth. It is possible to broaden the bandwidth by using a high-order LPF, although this would lead to phase lags. In the proposed system, force control with a low delay is achieved by using high-resolution rotary encoders and a 1st-order LPF.

The driving current reference 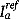 is obtained from the feedback of the compensation current 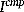 as:


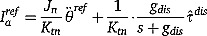


where *K*_*tn*_ is the nominal torque constant. The disturbance torque *τ*^*dis*^ is represented as:


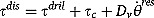


where *τ*^*dril*^ is the cutting torque, *τ*_*c*_ is the coulomb friction and 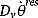 is the viscous friction. The coulomb friction *τ*_*c*_ can be neglected because the rotary motor is driven at high velocity. Then, the disturbance torque 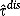 is represented as:


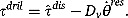


The estimated cutting torque *τ*^*dril*^ is obtained from the estimated disturbance torque 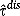 without the viscous friction 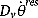. The viscous friction coefficient *D*_*v*_ is experimentally identified by the constant velocity test. When the velocity of the rotary motor is kept constant and the drill does not contact with the environment (no-load condition), the dynamics of the rotary motor is represented as:


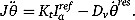


The estimated disturbance torque 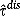 only consists of viscous friction. The viscous friction coefficient *D*_*v*_ is represented as:


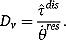


It is assumed that the non-linear friction of the linear motor is small enough to neglect. In addition, the non-linear friction is not taken into account because the rotational velocity is not near zero in the rotary system. However, identification and compensation of the non-linear friction are required for the multi-DOF system 25.

### Bilateral control

**Figure 4:**
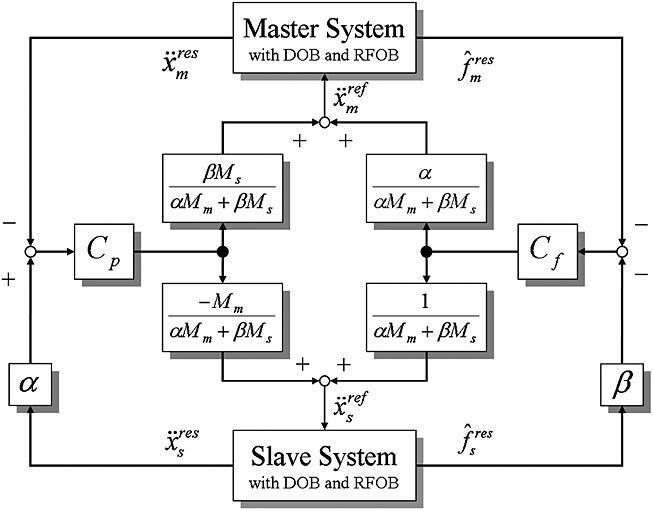
Acceleration-based four-channel bilateral control

A bilateral control system has been proposed. The four-channel bilateral control is a basic structure based on a two-port model that has four communication paths: one to transmit the master's motion (position/velocity); one to transmit the slave's motion (position/velocity); one to transmit the operating force to the master; and one to transmit the reaction force from the slave system generated by the contacted environment 16. Figure 4 shows the acceleration-based four-channel bilateral control system 17. *x* is the current position, *f* is the force, *C*_*p*_ is the position controller and *C*_*f*_ is the force controller. The subscripts *m* and *s* represent the variables of the master side and slave side, respectively. In the master–slave system with bilateral control, local disturbance is eliminated by the DOB. Thus, acceleration control is achieved at a cut-off frequency *g*_*dis*_ of the DOB. Additionally, the RFOB is applied to estimate the force from the master side response *f*_*m*_ and that from the slave side response *f*_*s*_. These force responses are given as estimated values 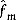 and 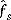 by the reaction force observer (RFOB). The targets of an ideal bilateral control system are position tracking and achieving the law of action and reaction between the master and slave systems. Position tracking by position control is obtained by:


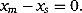


The artificial law of action and reaction by force control is obtained by:


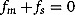


In addition, the four-channel bilateral control system achieves motion scaling. The goals of the scaled bilateral control are given as:


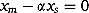



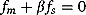


where *α* is a coefficient for position scaling and *β* is a coefficient for force scaling.

The position controller for the bilateral control system was a PD controller, which is obtained as:


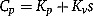


The force controller for the bilateral control system was a P controller, which is obtained as:


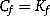


The differential control for achieving force control is not practical, because quantization noise effects are large in jerk. Position control is required for a fast and stable response. A controller can achieve zero steady-state error but then the response would not be as fast. For these reasons, the proposed force controller is a P-controlled one, and the proposed position controller is a PD-controlled one.

The acceleration references, which are inputs to the master and slave systems, are obtained from each controller and from the negative feedback of each response. The matrix of the equivalent mass **M** is defined by the target matrix of scaled bilateral control **J** as:


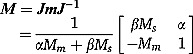



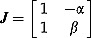



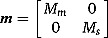


The acceleration references 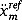 and 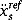 are obtained as:


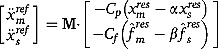


If the coefficient of position scaling *α* is > 1.0, then an enlarged motion is reproduced on the slave system. If the coefficient of force scaling *β* is > 1.0, then the operator can feel a minute change of force more clearly through the master system.

In a general bilateral control system, destabilization is caused by increasing the scaling coefficient 26. Sakaino *et al*. achieved decoupling of the scaled force control and scaled position control using oblique coordinate control 27. The scaled position control and the scaled force control act independently because the matrix of the equivalent mass prevents the interference between these controls.

The controller was implemented by using the real-time operating system RT-Linux 3.2. The sampling period of the real-time task by RT-Linux was set as 0.1 ms. The delay between each channel is extremely small, since the control period is short. Therefore, it is assumed that system destabilization caused by the network delay 28–29 and the control period 30 will not occur.

### Experimental set-up

**Figure 5:**
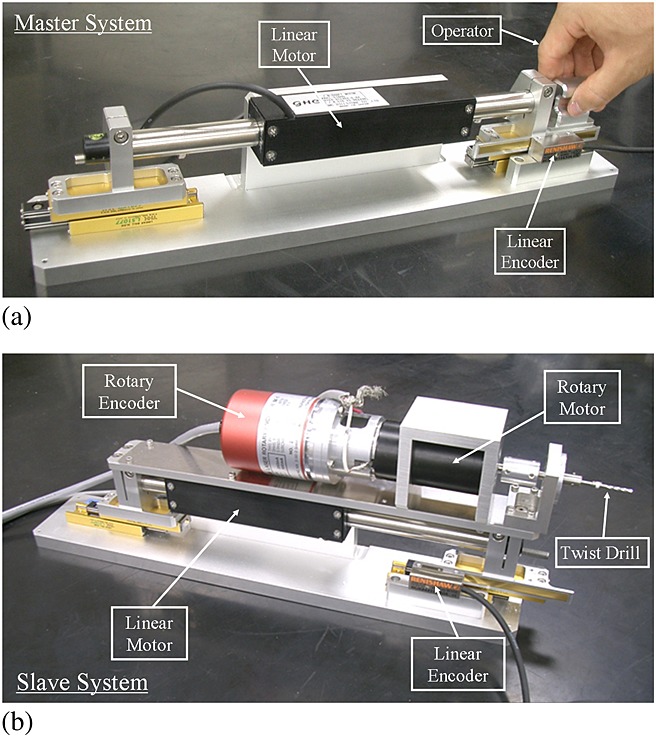
Overview of the proposed system: (a) master system; (b) slave system

**Figure 6:**
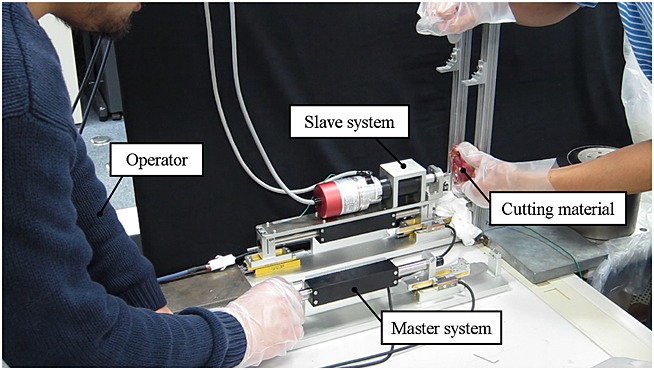
Experimental setting

The details of the master and slave systems used in the experiment are shown in Figure 5. Figure 6 shows this experimental setting. A human operator directly manipulates the master system. The estimated cutting torque and cutting force is graphically displayed on the PC monitor in real time, and allows the surgeon to sense the applied force accurately. At the same time, hazard detection and sharing of the force sensation with other surgeons are achieved.

The parameters of the rotary system are listed in Table 1 and the parameters of the feed system in Table 2. The angular velocity command is set at 900 rpm, which is the general rotational speed used in the drilling of the bone tissue in dental implant surgeries.

**Table 1:** Parameters of rotary system

Thrust coefficient, *K_tn_*	0.0603 N/A
Nominal inertia, *J_n_*	0.000138 kg/m^2^
Resolution of rotary encoder	200000 pulse/rev
Angle gain, *K_a_*	120
Angular velocity gain, *K_av_*	50
Force gain, *K_f_*	1
Cut-off frequency of pseudo-derivation, *g_pd_*	640 rad/s
Cut-off frequency of DOB, *g_dis_*	640 rad/s
Cut-off frequency of RTOB, *g_reac_*	640 rad/s

**Table 2:** Parameters of feed system

Thrust coefficient, *K_tn_*	22.0 N/A
Nominal mass (master), *M_m_*	0.5 kg
Nominal mass (slave), *M_m_*	1.2 kg
Resolution of linear encoder	0.0001 mm
Position gain, *K_p_*	1600
Velocity gain, *K_v_*	80
Force gain, *K_f_*	1
Cut-off frequency of pseudo-derivation, *g_pd_*	800 rad/s
Cut-off frequency of DOB, *g_dis_*	800 rad/s
Cut-off frequency of RFOB, *g_reac_*	800 rad/s

Under the critically damped system, the relationship between the position gain *K_p_* and velocity gain *K_v_* is obtained as:


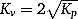


The *K_p_* and *K_f_* should be large, so as to improve the reproducibility and operationality of the system. In the proposed system, *K_p_* and *K_f_* are kept at the highest values possible in this experiment.

**Figure 7:**
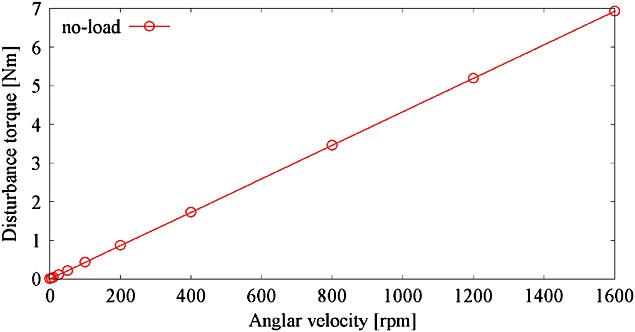
Constant velocity test

Figure 7 shows the experimental results of the constant velocity test for identification of the viscous friction coefficient in the case of the rotary system. The viscous friction coefficient is determined experimentally to be *D_v_* = 0.0043 Nm/rpm. In the case of the feed system, the friction force of the linear motor is small enough and hence can be neglected. Therefore, the constant velocity test was not conducted for the feed system.

## Results

In this experiment, the operation to achieve perforations in the bone was performed twice. In the first operation, the scaling coefficients were set as *α* = 1.0 and *β* = 1.0. The angular velocity control of the rotation of the drill and bilateral control of the master–slave system were started at 0 s. At this time, the initial distance between the drill tip and the bone was set close. The operator started cutting from about 1.2 s. The drill tip went through the cutting material in about 9.9 s. Finally, the drill was pulled out and returned to its initial position. In the second operation, the scaling coefficients were changed to *α* = 2.0 and *β* = 2.0 after 15.0 s. The operator started the cutting from about 18.2 s. The second perforation occurred at about 31.2 s.

**Figure 8:**
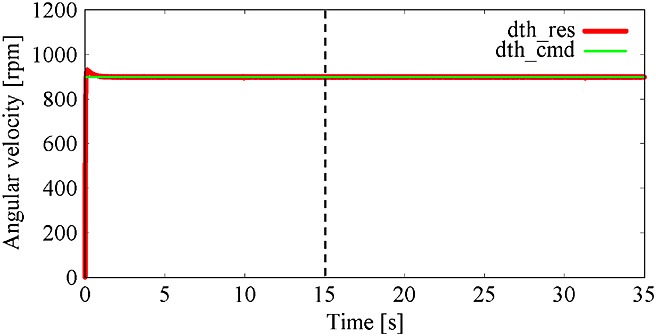
Angular velocity response

**Figure 9:**
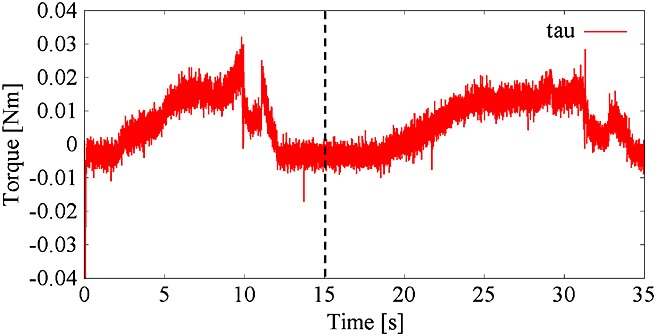
Estimated cutting torque

**Figure 10:**
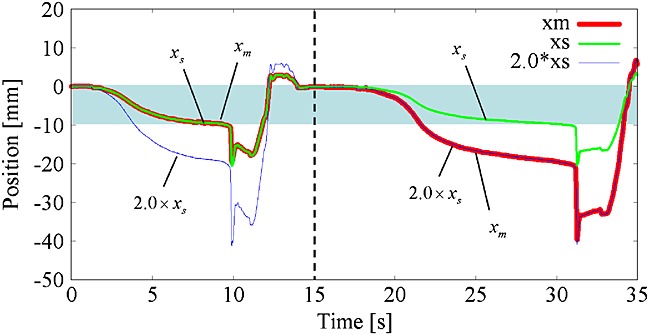
Position response

**Figure 11:**
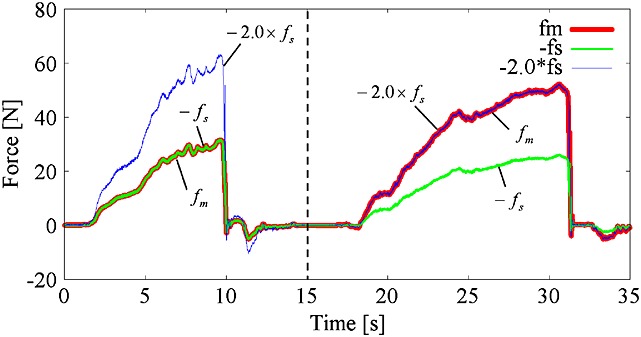
Force response

**Figure 12:**
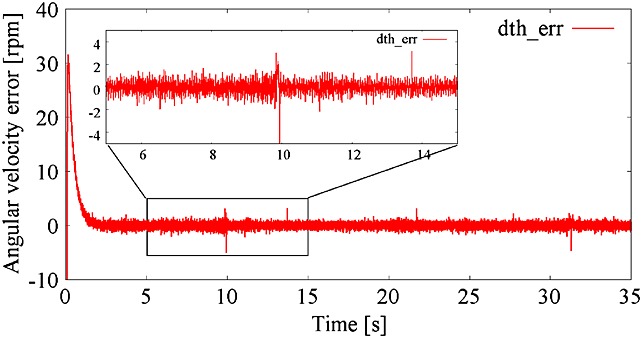
Angular velocity error

**Figure 13:**
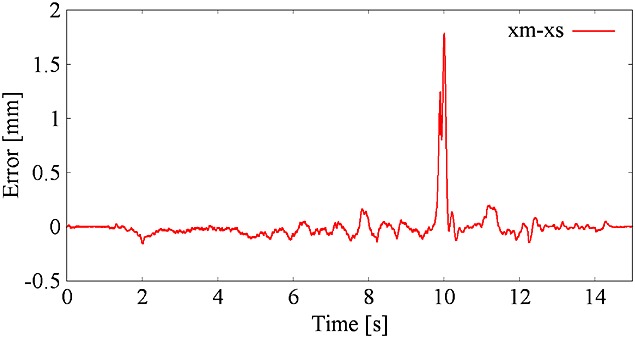
Tracking error of the master–slave system

**Figure 14:**
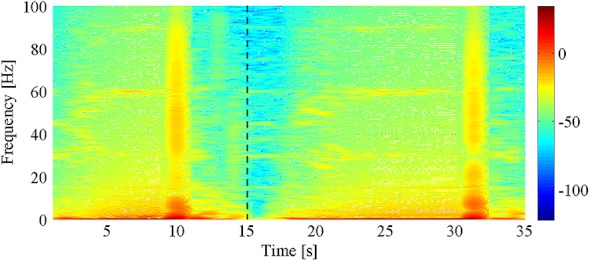
Spectrogram of force response

Figure 8 shows the results of the angular velocity response. Figure 9 shows the results of the estimated cutting torque. Figure 10 shows the results of the position response in the feed direction. In Figure 10, the blue-shaded area represents the thickness of the cutting material. Figure 11 shows the results of the force response in the feed direction. Figure 12 shows the error of angular velocity 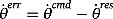. Figure 13 shows the tracking error *x*^*err*^ = *x*_*m*_ − *x*_*s*_ between the position of the master system and that of the slave system. Figure 14 shows the spectrogram of the force response 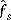. In order to visualize the cutting vibration and the cutting phenomenon, short-time Fourier transform (STFT) is used. STFT is the time–frequency analysis, which is used for one of the image-processing techniques 31.

## Discussion

The performances of angular velocity regulation and position tracking and the reproducibility of force transmission are evaluated here. As seen in Figure 8, the angular velocity response was regulated to the angular velocity command of 900 rpm, irrespective of whether the drill tip was or was not in contact with the cutting material. As seen in Figure 9, real-time monitoring of cutting torque was achieved.

From Figure 10, it was confirmed that the position of the slave was tracked to the position of the master system. Therefore, the operator's motion was reproduced at the slave side. Under the effect of scaled bilateral control, position tracking was achieved between the master system and the virtually enlarged slave system. The feed operation was scaled down in the slave system by adjusting the position scaling coefficient *β*. As a result, a more precise feed motion was achieved.

From Figure 11, it was confirmed that the artificial law of the action and reaction was achieved between the master and the slave systems. The operator experienced the cutting force from the master system during the drilling process. Under the effect of scaled bilateral control, the cutting force was enlarged and transmitted to the operator. The operator could clearly feel the change in cutting force when the perforation occurred.

As seen in Figure 12, the error was suppressed to < ± 2.0 rpm during the drilling process. When the perforation occurred, the maximum error was −5.0 rpm. The overshoot and the settling time can be adjusted by the controller gains according to the required specification.

As shown in Figure 13, the tracking error was suppressed to < ± 0.2 mm during the drilling process. When the perforation occurred, the maximum error was −1.8 mm. If position scaling is applied, this error range is enlarged, depending on the scaling coefficient *α*. Therefore, in order to determine an ideal scaling coefficient, it is necessary to take into account the error range that satisfies the requirement specification for manipulation accuracy.

From Figure 14, the frequency of the cutting vibration can be theoretically defined as:


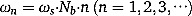


where *ω*_*n*_ is the frequency of the cutting vibration, *ω*_*s*_ is the spindle frequency of drill rotation, and *N*_*b*_ is the number of blades. In this experiment, the spindle frequency *ω*_*s*_ was 15 Hz because the angular velocity command was set at 900 rpm. The twist drill had two blades, *N*_*b*_ = 2. Therefore, the theoretical frequencies of the cutting vibration are determined to be *ω*_1_ = 30 Hz, *ω*_2_ = 60 Hz and *ω*_3_ = 90 Hz. From Figure 14, it can be seen that each peak was found in the spectrogram at 30, 60 and 90 Hz. The peaks were consistent with the theoretical frequencies. Therefore, it was proved experimentally that the transmission of the cutting vibration to the operator in the high-frequency band was achieved.

## Conclusion

In this study, a telerobotic-assisted drilling system was proposed for dental implant surgery. The proposed system is composed of a master system and a slave system with acceleration-based bilateral control. Position tracking and law of action and reaction were achieved between the master and slave systems by acceleration-based four-channel bilateral control. The performances of the proposed system were evaluated experimentally. From these results, it was shown that it is possible to transmit cutting force and vivid vibration to the operator. The force sensorless monitoring of the cutting force is achieved by RTOB and RFOB. The precise feed operation of the drilling process is achieved by position scaling and force scaling.
